# Comparison of the Legal Infrastructure Governing Psychiatric Practice and Its Implementation in Tanzania and the UK

**DOI:** 10.1192/bjo.2024.215

**Published:** 2024-08-01

**Authors:** Jessica Morgan

**Affiliations:** Stoke Mandeville Hospital, Buckinghamshire Healthcare Trust, Aylesbury, United Kingdom. University of Oxford, Oxford, United Kingdom

## Abstract

**Aims:**

To examine the legal framework governing psychiatric practice, specifically focusing on the Mental Health Act, within a singular psychiatric centre in Moshi, Tanzania. The primary objective was to explore the intersection of culture and legalities in shaping ethical practice within this emerging unit. Drawing on a comparative analysis of contents and the implementation of the MHA in Tanzania and in the UK, the study aims to understand the ways in which cultural contexts influence the legal and ethical dimensions of psychiatric care.

**Methods:**

This was a multi-method study that combined literature analysis, structured interviewing, and structured reflective practice.

1. Direct comparison of the UK and Tanzanian MHA.
2. Evaluation of clinician understanding of the MHA through structured interviewing of clinicians with respect to their knowledge of the MHA, its existence, and its key components.
3. Analysis of implementation of the MHA in liaison psychiatry in both centers. Compared through unstructured interviewing and reflective practice.

**Results:**

1. The most striking difference is the length of the documents. The Tanzanian MHA is 27 pages while the UK MHA is 173. This additional length covers: Admission and Discharge Procedures, explanation of the Roles and Powers of Professionals, and further discussion on Safeguards and Rights of Individuals.
2. When interviewed, only 15% of Tanzanian physicians could explain what the MHA is, compared with 100% of UK physicians (N = 40).
3. In the UK, all doctors use the MHA and implement DOLS. In Tanzania, this falls under the role of liaison psychiatrists. This is likely because, the MHA is included in the UK's medical curricula but not in Tanzania's.

**Conclusion:**

Lack of understanding of the MHA and other key laws in psychiatry is a global issue, not limited to the UK or Tanzania. However, physicians with strong understanding are more scarce in Tanzania. This scarcity puts additional pressures on psychiatric services, as psychiatrists are called to assess issues of capacity or consent that could be assessed by any doctors in the UK. However, this means that the MHA and MCA are almost solely used by psychiatrists and therefore often assessed to a very high standard. It must be considered that on reflection, I have also observed physicians with limited understanding of the MHA, capacity and consent within the NHS. Imposing a higher standard on another culture would be unethical. Efforts into educating medical students and professionals is required in the UK and Tanzania.

